# Reduced electron/hole recombination in Z-scheme nanostructure of zeolitic imidazolate framework-11/graphitic carbon nitride as photocatalyst under visible light

**DOI:** 10.1038/s41598-023-49315-7

**Published:** 2023-12-18

**Authors:** Goli YarAhmadi, Narjes Keramati

**Affiliations:** Department of Nanotechnology, Faculty of New Sciences and Technologies, Semnan University, Semnan, Iran

**Keywords:** Environmental sciences, Chemistry, Engineering

## Abstract

In this research, for the first time, the synthesis of nanostructure of zeolitic imidazolate framework-11/graphitic carbon nitride (ZIF-11/g-C_3_N_4_ X) with different weight of g-C_3_N_4_ (X: 0.01, 0.1, 0.3 g) is reported. Their performance was compared in photocatalytic degradation of MB under visible light. Synthetic samples were characterized by X-Ray diffraction (XRD), Fourier-transform infrared spectroscopy (FTIR), X-ray photoelectron spectrometer (XPS), diffused reflectance spectroscopy (DRS), Field emission scanning electron microscopy (FE-SEM), Transmission electron microscope (TEM), Brunauer–Emmett–Teller (BET), Electrochemical Impedance Spectroscopy (EIS) and Photoluminescence (PL) analysis. Based on the results, Z-scheme ZIF-11/g-C_3_N_4_ 0.3 was selected as the best sample. FESEM and TEM images indicated that g-C_3_N_4_ sheets were complicated on the surface of ZIF-11 with rhombic dodecahedron (RHO) morphology. The surface area and band gap of ZIF-11/g-C_3_N_4_ 0.3 was determined as 174.5 m^2^/g and 2.58 eV, respectively. The recombination of charge carriers in the ZIF-11/g-C_3_N_4_ 0.3 nanostructure was reduced. Photocatalytic degradation efficiency of MB (5 ppm), pH = 7, visible irradiation (120 W-60 min) using 0.1 g of ZIF-11/g-C_3_N_4_ 0.3 was achieved 72.7% with first-order kinetic model and acceptable stability in three consecutive cycles. Further, the total organic carbon (TOC) removal rate by ZIF-11/g-C_3_N_4_ 0.3 after 5 h were 66.5%.

## Introduction

In recent years, various dyes are used in different industries such as textiles, food, rubber, printing, cosmetics, medicine, plastic, concrete, and the paper industry for multiple purposes. These industries generate a tremendous amount of wastewater containing carcinogenic and toxic dyes that pollute water, which becomes unfit for human consumption. Among these industries, the textile industry is the most dye-consuming industry utilizing textile dyes, which are highly complex compounds with different structural groups. One of the highest-consuming materials in the dye industry is methylene blue (MB), which is commonly used for coloring silk, wool, cotton, and paper. Excessive discharge of it is toxic and carcinogenic to the environment. MB discharge into the environment is a significant threat for aesthetical and toxicological reasons. It also reduces light penetration and is a toxic supply to food chains for organisms^1,2^. Many methods such as adsorption^3–5^, reverse osmosis, coagulation, liquid–liquid extraction and flocculation have been used to overcome this serious problem of water pollution^1^. Due to the thermal and light stability and non-biodegradability, it is tough to degrade MB dye into smaller inorganic molecules by employing common methods^1^. As one of the advanced environmental purification technologies, the photocatalytic degradation has attracted more attention in creating an option for the problems of dyes pollution^3–11^. Existence of an ideal photocatalyst that can effectively absorb light and use the photo-generated charge carriers for the reduction reaction is still a challenge^3^.

Zeolitic Imidazolate Frameworks (ZIFs) are crystalline compounds that are of great interest due to their unique physical, chemical and optical properties and their high surface-to-volume ratio^12–14^. In other hand, graphitic carbon nitride (g-C_3_N_4_) as a metal-free polymer semiconductor, due to the activity under visible light with a suitable band gap, non-toxicity, high chemical stability, and simple preparation methods being convenient and inexpensive is known as one of the selected photocatalytic materials^14–19^. Pairing suitable photocatalyst is considered as one of the most ideal approaches to facilitate the separation of the photo-generated electrons and holes and improve photocatalytic activities^12,20^.

Yuvan et al. made a set of ZIF-8/g-C_3_N_4_ photocatalysts with high response under visible light using in situ ultrasonic method^21^. Its photocatalytic activity has been investigated by degrading Methyl Orange (MO), Rhodamine B (RhB) and Tetracycline (TC). Due to the formation of heterogeneous photocatalyst and the normal type II heterogeneity transmission mechanism, the reduction of recombination and the transfer of the absorption edge to the visible light region have finally increased the performance of the mentioned photocatalyst^21^. Wang et al. have synthesized g-C_3_N_4_/MOF (ZIF-67) photocatalyst using hydrothermal method and evaluated its photocatalytic activity by producing hydrogen under visible light irradiation^22^. The photocatalytic activity of H_2_ production was 30 times higher than pure g-C_3_N_4_, because the specific surface area has been significantly increased, therewith, it has inhibited electron/hole recombination^22^.

Zeolitic imidazolate framework-11 (ZIF-11) with dodecahedron (RHO) morphology is one of the most auspicious zeolitic imidazolate framework for gas separation and adsorption^14,23^ due to its huge porosity, and it has large cavities connected with small pore apertures. The aim of the current research is to fabricate zeolitic imidazolate framework-11/graphitic nitride carbon nanostructure (ZIF-11/g-C_3_N_4_) for the first time and investigate its photocatalytic performance in the degradation of MB under visible light irradiation. The characterizations of the materials were obtained by using different techniques, and the possible degradation pathway and mechanism were also proposed. According to the photocatalytic performance of synthesized ZIF-11/g-C_3_N_4_ composite on degradation of MB, the charge transfer mechanism of Z-Scheme and reduction of electron/hole recombination was achieved, which are suitable for wastewater treatment.

## Experimental

### Materials

Toluene (C_6_H_5_CH_3_), Ethanol (C_2_H_5_OH), Sulfuric Acid (H_2_SO_4_), Benzimidazole (C_7_H_6_N_2_), Zinc Acetate Dihydrate (C_4_H_10_O_6_Zn·2H_2_O), Urea (C_6_H_11_NO_4_), Ammonium Hydroxide (NH_4_OH), Hydrochloric Acid (HCl), Sodium Hydroxide (NaOH), Sodium Chloride (NaCl), Methanol (CH_3_OH) and Methylene Blue (C_16_H_18_ClN_3_S) were purchased from Merck company. All chemicals used in this work were of analytical grade, obtained from commercial supplier, and used without further purification.

### Synthesis of zeolitic-11 imidazolate framework

ZIF-11 micro particle was synthesized based on our previous research with slight modifications^14^. First, 0.12 g of benzimidazole was dissolved in 4.8 g of methanol, along with 4.6 g of toluene and 1.2 g of ammonium hydroxide under continuous stirring. Then, 0.11 g of zinc acetate was added and was continuously stirred at ambient temperature for 3 h. After that, the solid was separated and washed three times with methanol to completely remove the toluene. Finally, it was dried at room temperature for 12 h. The synthetic sample was named as ZIF-11.

### Synthesis of graphitic carbon nitride

The g-C_3_N_4_ powder was prepared by thermal polymerization similar to our previous research work^2^. First, 16 g of urea was heated in a 100 mL aluminum crucible with a lid for 4 h at 550 °C by 2 °C/min. Finally, a yellow powder product has been obtained. The synthetic sample was named as g-C_3_N_4_.

### Synthesis of zeolitic-11 imidazolate framework/graphitic carbon nitride

**Figure 1:**
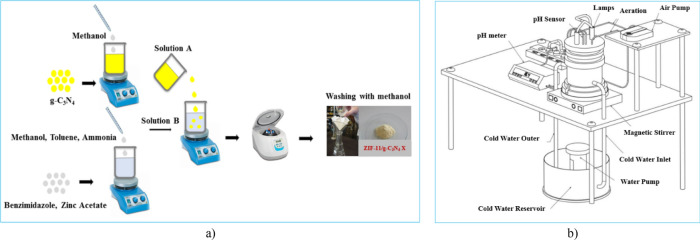
(**a**) Schematic illustration of synthesis process of g-C_3_N_4_/ZIF-11 X, (**b**) schematic representation of the homemade photo-reactor.

Zeolitic imidazolate framework-11/graphitic carbon nitride was synthesized by a simple method at room temperature. First, a certain amount of synthesized g-C_3_N_4_ was dispersed in 6.1 mL methanol for 150 min (solution A). Then, another solution was prepared by dissolving 0.12 g of benzimidazole in 6.1 mL methanol, 5.3 mL toluene and 0.8 mL ammonia and finally adding 0.11 g of zinc acetate (solution B). Afterward, solution A was added to solution B and stirred for 3 h at room temperature. Then, the solid was separated and washed three times with methanol. Finally, it was dried at room temperature for 3 h (Fig. 1a). The synthetic samples were named as ZIF-11/g-C_3_N_4_ X. The value of X was the weight of g-C_3_N_4_ (0.01, 0.1, and 0.3 g) which was used in the synthesis.

### Characterization

The crystal structure of the material was determined by X-ray diffraction (D8-Advance, Bruker, Germany) using Cu Kα radiation (λ = 1.5406 Å) in the 2θ range from 10° to 80°. Fourier transform infrared spectroscopy (FTIR, SHIMADZU 8400S, Japan) has been used to study the structure and chemical bonds of the molecule. X-ray photoelectron spectrometer (XPS) with a monochromated AlKα source at a power of 180 W (Specs/FlexPS, Germany). The surface morphology of the sample was observed with a field emission scanning electron microscope (FESEM, ZISS). Energy dispersive X-ray spectroscopy (EDS) (MIRA III SAMX detector, France) was performed to determine the percentage of elements in the composition. The transmission electron microscope (TEM) image was obtained from a Phillips model EM208S device. Nitrogen adsorption–desorption isotherms (BELSORP MINI II, BEL company) were recorded with Quantachrome at liquid N_2_ temperature. The specific surface area was determined from the linear part of the BET diagram. The light absorption properties of solid samples were determined by UV–visible DRS (Avaspec-2048-TEC, Netherlands). In order to investigate the degree of electron/hole separation, photoluminescence (PL) spectrum (Avaspec-2048-TEC, Netherlands) and electrochemical impedance (EIS) (Sharif solar co, model PGE-18) were determined.

### Photocatalytic performance

The photocatalytic degradation of MB in an aqueous medium was investigated in a homemade Pyrex photo-reactor in batch mode (Fig. 1b).

In this way, the photocatalytic activity of the synthetic samples was investigated using a 200 mL of MB solution (5 ppm) using 0.1 g of synthesized photocatalyst under visible light irradiation (120 W). For this purpose, MB must reach the adsorption/desorption equilibrium on the surface of the photocatalyst, so the catalyst was exposed to dark for 60 min, then lamps was turned on and after 60 min of irradiation, the photocatalyst was separated from the solution by centrifugation. The efficiency of photocatalytic degradation of MB (D %) by the synthesized samples was evaluated by measuring the decrease in the intensity of the absorption band (%D = $\frac{{C}_{0}-C}{{C}_{0}}$) at the wavelength of 664 nm by UV–Vis spectrophotometer. The concentration of MB at illumination time t was recorded as C.

### Optimization of photocatalytic degradation of MB

In order to investigating the effective factors on the process, optimization and interaction between them, the design of the experiment was carried out. Response surface methodology (RSM) is one of the mathematical and statistical methods, a common method of which is central composite design (CCD), to build experimental models of the studied process^10,11,24^. Three operational parameters of the degradation process (photocatalyst concentration, initial pH value of the solution and irradiation time) were optimized by the RSM method. The photocatalytic degradation efficiency of MB (with a constant concentration of 10 ppm in all statistical experiments) was selected as the response. By performing preliminary tests, the levels of selected factors were determined and listed in Table 1. Calculations related to the design of experiments were performed by Design Expert 11 software.

**Table 1:** Selected levels of operational parameters for photocatalytic degradation of MB under visible light.

Parameter	Low level (− 1)	High level (+ 1)
A: Irradiation time (min)	60	120
B: Photocatalyst concentration (g/L)	0.5	1.5
C: pH	4	10

## Results and discussion

### Characterization

**Figure 2:**
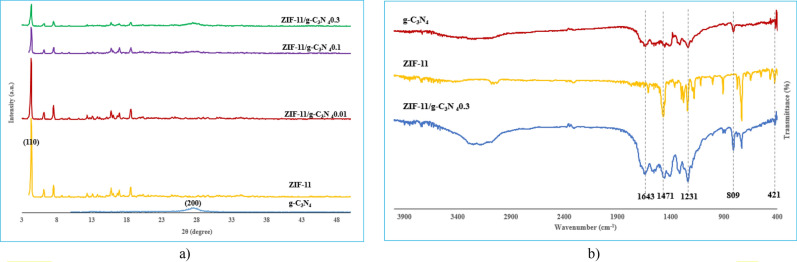
(**a**) XRD pattern of g-C_3_N_4_, ZIF-11, ZIF-11/g-C_3_N_4_0.01, ZIF-11/g-C_3_N_4_0.1, ZIF-11/g-C_3_N_4_0.3, (**b**) FTIR spectrum of ZIF-11, g-C_3_N_4_, ZIF-11/g-C_3_N_4_0.3

Crystal structure and phase purity of synthesized samples (g-C_3_N_4_, ZIF-11, ZIF-11/g-C_3_N_4_0.01, ZIF-11/g-C_3_N_4_0.1 and ZIF-11/g-C_3_N_4_0.3) was studied by XRD and shown in Fig. 2a. According to the XRD pattern of g-C_3_N_4_ sample, it has two characteristic diffraction peaks at 27.3° and 13.4° corresponding to (200) and (001) planes, which confirms the crystal structure (JCPDS No. 87-1526)^2,21^. In the ZIF-11 sample, the sharp peak had appeared at 4.3° belonging to the (110) plane (CCDC No. 602545)^14^. The characteristic peaks of ZIF-11 and g-C_3_N_4_ have also appeared in all composites. In the ZIF-11/g-C_3_N_4_0.01 sample, the peak of g-C_3_N_4_ index was not observed due to its low content in the structure, and with the increase in the amount of g-C_3_N_4_, the intensity of its index peak also increased. The crystallite size using the Debye-Scherer equation for g-C_3_N_4_, ZIF-11, ZIF-11/g-C_3_N_4_0.1, ZIF-11/g-C_3_N_4_0.3 were calculated as 20, 26.9, 26.5, 22.4 and 22.4 nm, respectively.

The FTIR spectra of three samples g-C_3_N_4_, ZIF-11 and ZIF-11/g-C_3_N_4_0.3 have been investigated using FTIR spectroscopy (Fig. 2b). According to spectrum of ZIF-11, the peak that occurs at 421 cm^−1^ was related to Zn-N stretching. Peaks at 1471 and 1610 cm^−1^ were attributed to C–C stretching in the aromatic benzimidazole ring, and peaks in the region of 600–1500 cm^−1^ were related to total stretching or bending of the benzimidazole ring^14,25^. Three main regions for g-C_3_N_4_ can be seen. A sharp peak corresponds to 3-S triazine units was observed at 809 cm^−1^. The characteristic peaks at 1231, 1327, 1417 and 1573 cm^−1^ were assigned to the N–C stretching vibrational state of aromatic rings, the peak at 1643 cm^−1^ was assigned to the N=C stretching vibrational state. Broad absorption peaks in the range of 3100 to 3300 cm^−1^ are related to the stretching vibrations of amines^2^. All mentioned characteristic peak were appeared in ZIF-11/g-C_3_N_4_0.3 composite.

**Figure 3:**
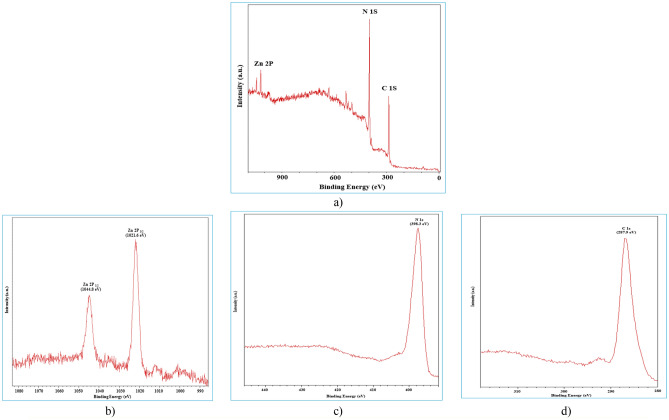
XPS survey spectra of (**a**) ZIF-11/g-C_3_N_4_0.3 composite and high-resolution XPS spectra of (**b**) Zn 2p, (**c**) N 1 s, (**d**) C 1 s.

XPS was applied for detecting the composition of the samples. The results are shown in Fig. 3. According to the survey spectra shown in Fig. 3a, it can be observed that ZIF-11/g-C_3_N_4_0.3 composite are composed of C, N and Zn elements. The high-resolution XPS spectra of Zn 2p are shown in Fig. 3b. The two peaks at binding energy of 1021.6 eV and 1044.8 eV in ZIF-11/g-C_3_N_4_0.3 ascribed to Zn 2p_3/2_ and Zn 2p_1/2_, respectively. Figure 3c shows the C 1 s high-resolution XPS spectrum of ZIF-11/g-C_3_N_4_0.3 shown in Fig. 3d, dominant peak at 287.9 eV corresponded to N=C–N bonds. The N 1 s spectrum of ZIF-11/g-C_3_N_4_0.3, with peak at binding energy of 398.3 eV which was derived from sp2 hybridized aromatic nitrogen atoms bound to carbon atoms (C=N–C).

**Figure 4:**
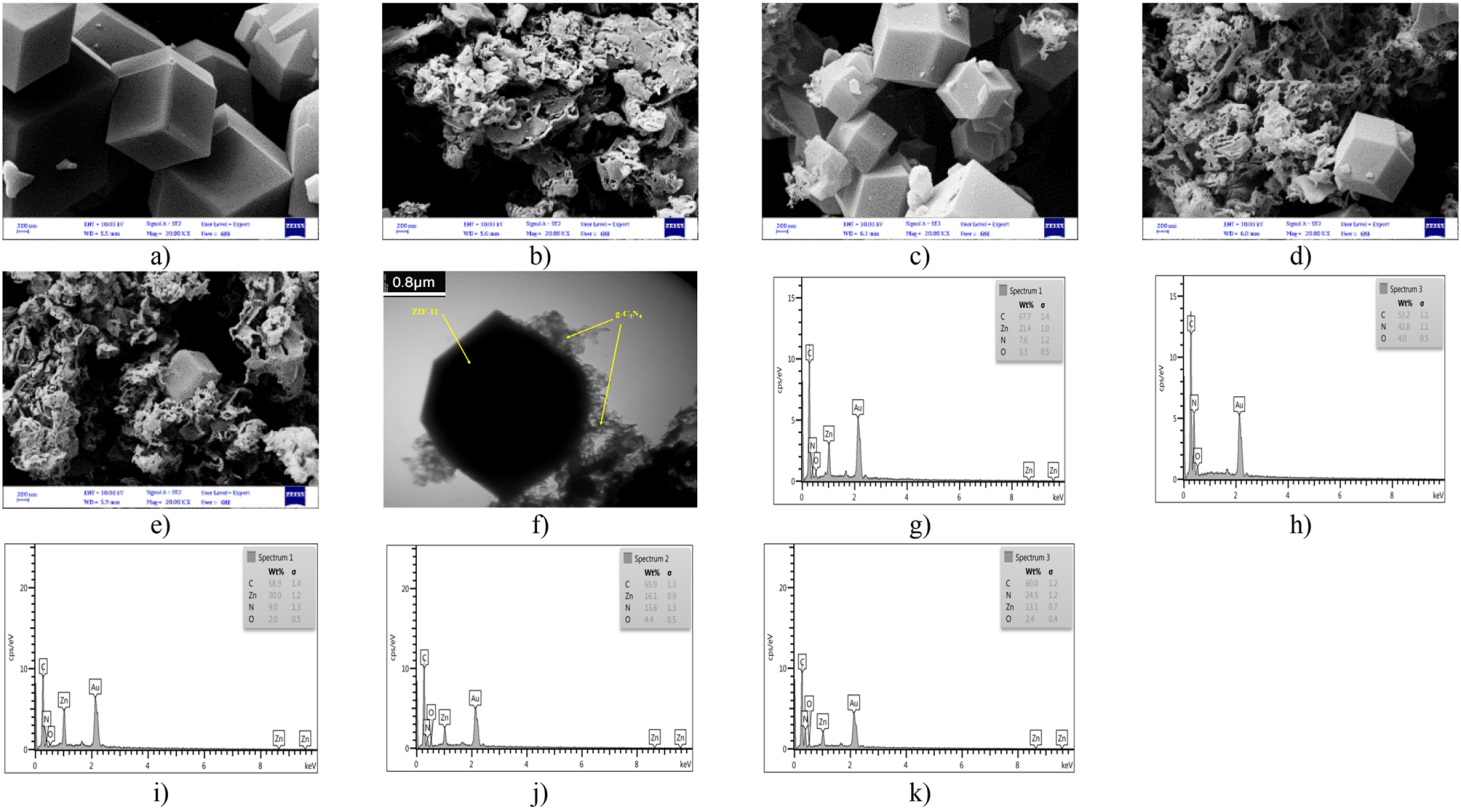
FESEM images of (**a**) ZIF-11, (**b**) g-C_3_N_4_, (**c**) ZIF-11/g-C_3_N_4_0.01, (**d**) ZIF-11/g-C_3_N_4_0.1, (**e**) ZIF-11 11/g-C_3_N_4_0.3, (**f**) TEM image of ZIF-11/g-C_3_N_4_0.3, EDX spectrum of (**g**) ZIF-11, (**h**) g-C_3_N_4_, (**i**) ZIF-11/g-C_3_N_4_ 0.01, (**j**) ZIF-11/g-C_3_N_4_ 0.1, (**k**) ZIF-11 11/g-C_3_N_4_ 0.3

The morphology of all synthesized samples was observed by FESEM (Fig. 4a–e), which shows that the as-synthesized ZIF-11 samples have a dodecahedron structure^14,25^ while g-C_3_N_4_ shows a thin sheet-like structure^2^. The images of ZIF-11/g-C_3_N_4_ composites (Fig. 4c–e) show the RHO morphology of ZIF-11 on the rough surface of g-C_3_N_4_. It was found that the g-C_3_N_4_ sheets surrounded ZIF-11 crystals from different directions and, of course, kept the crystal structure of ZIF-11. Next, in Fig. 4f, TEM was also used further ZIF-11/g-C_3_N_4_0.3 morphology. As shown in Fig. 4f, ZIF-11 reveals a RHO assembly whereas g-C_3_N_4_ shows a thin sheet-like structure which can be attached to the ZIF-11 surface. Combined with SEM, TEM results prove that g-C_3_N_4_ successfully attached to the ZIF-11. The EDS analysis (Fig. 4g–k) confirmed the correctness of the synthesis of pure samples.

**Figure 5:**
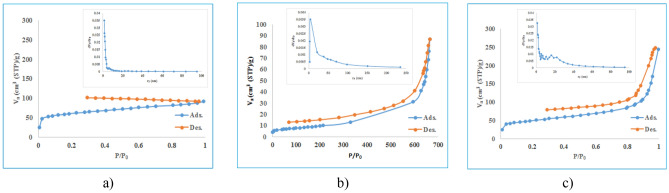
Nitrogen adsorption/desorption isotherm and BJH analysis (**a**) ZIF-11, (**b**) g-C_3_N_4_, (**c**) ZIF-11/g-C_3_N_4_ 0.3

N_2_ adsorption–desorption isotherm was investigated in order to obtain more information about the specific BET surface areas and the pore structure of the samples. The results are shown in Fig. 5. Also, the specific surface area, pore volume and pore size data of samples are listed in Table 2. As shown, the ZIF-11/g C_3_N_4_0.3 sample shows a higher specific surface area than pure g-C_3_N_4_, which is due to the contribution of ZIF-11, which has a large specific surface area in the structure. Increasing the surface area by introducing of MOF was reported in previous study^26^. All samples have pore diameters in a range of 2–50 nm, therefore, they have mesoporous structure.

**Table 2:** Specific surface area and pore characteristics of synthetic samples.

Sample	Surface area (m^2^/g)	Pore volume (cm^3^ g^−1^)	Pore size (nm)
ZIF-11	219	0.14	2.6
g-C_3_N_4_	30.2	0.15	9.8
ZIF-11/g-C_3_N_4_ 0.3	174.5	0.36	8.2

**Figure 6:**
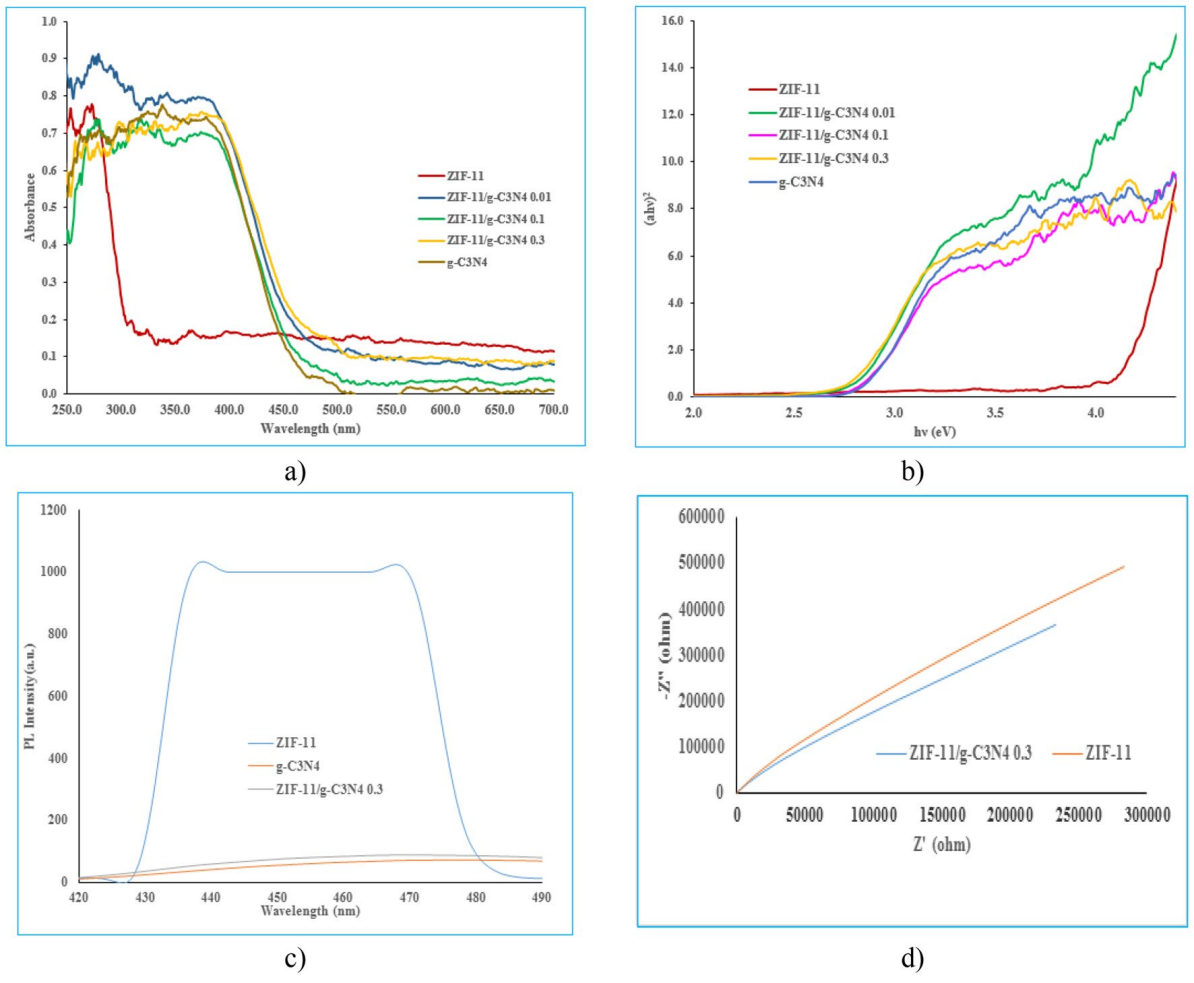
(**a**) UV–Vis DRS of ZIF-11, g-C_3_N_4_, ZIF-11/g-C_3_N_4_ 0.01, ZIF-11/g-C_3_N_4_ 0.1 and ZIF-11/g-C_3_N_4_ 0.3, (**b**) Tauc plots, (**c**) PL spectra of ZIF-11, g-C_3_N_4_ and ZIF-11/g-C_3_N_4_ 0.3, (**d**) EIS diagram of ZIF-11 and ZIF-11/g-C_3_N_4_0.3

Figure 6a shows the UV–Vis absorption spectrum of g-C_3_N_4_, ZIF-11, ZIF-11/g-C_3_N_4_0.01, ZIF-11/g-C_3_N_4_0.1 and ZIF-11/g-C_3_N_4_0.3 photocatalysts. All samples have the ability to be activated under visible and ultraviolet light. The band gap value of them were calculated by Tauc plot (Fig. 6b) as 2.64, 4.1, 2.60, 2.64 and 2.58 eV, respectively. The PL of g-C_3_N_4_, ZIF-11 and ZIF-11/g-C_3_N_4_0.3 samples was investigated to investigate the transfer process, separation efficiency and electron/hole pair recombination rate caused by light irradiation (Fig. 6c). Obviously, after coupling ZIF-11 by g-C_3_N_4_, the intensity of PL emission decreases significantly, which indicates the effective separation of charge carriers and reduction of the electron/hole recombination rate in ZIF-11/g-C_3_N_4_0.3 heterogeneous photocatalyst compared to pure ZIF-11. This can indicate that there are strong interactions between ZIF-11 and g-C_3_N_4_ and the formation of an interface between ZIF-11 and g-C_3_N_4_, which is useful for surface charge transfer. Finally, this lack of electron–hole recombination photo-generated increases the pollutant degradation efficiency^27^. EIS analysis was also performed to describe the electrical properties of the materials in order to evaluate the separation efficiency of charge carriers. The smaller arc radius is for more effective separation of electron/hole pairs created by light^28^. According to Fig. 6d, the semicircle of ZIF-11/g-C_3_N_4_0.3 is smaller than ZIF-11 in the high frequency region, which indicates that ZIF-11/g-C_3_N_4_0.3 has a lower resistance than ZIF-11, which causes better carrier transfer. Therefore, ZIF-11/g-C_3_N_4_0.3 has a stronger thermodynamic driving force for the photocatalytic degradation of MB.

### Photocatalytic degradation of MB under visible light irradiation

The photocatalytic degradation efficiency of MB (MB: 5 ppm, pH: 7, Photocatalyst: 0.1 g, 120 W visible light irradiation, time: 60 min) was investigated and compared by ZIF-11, g-C_3_N_4_, ZIF-11/g-C_3_N_4_ 0.01, ZIF-11/g-C_3_N_4_ 0.1 and ZIF-11/g-C_3_N_4_ 0.3, which was obtained 16.7, 25.9, 70.6, 72.7 and 51.2%, respectively.

Among composites, ZIF-11/ g-C_3_N_4_0.01 has the lowest degradation efficiency and ZIF-11/g-C_3_N_4_0.3 sample has the highest, and all of them have shown better performance than pure ZIF-11 and g-C_3_N_4_. The synergistic effect of the two components in improving the ability to absorb visible light, as well as in accordance with the PL spectrum, the effective charge transfer between the two components and subsequently the reduction of electron/hole recombination has led to higher destruction efficiency. Based on the obtained results, the ZIF-11/g-C_3_N_4_0.3 sample was selected as the best synthetic sample and in following, optimization of degradation was done by using it.

With the aim of investigating the effective factors on the process, the interactions between them and their optimization, an experimental design based on CCD for the photocatalytic degradation of MB was carried out using ZIF-11/g-C_3_N_4_ 0.3 sample. The results of the design are presented in Table 3.

**Table 3:** The design matrix of the central complex; factors, levels, experimental responses.

Test number	A: Irradiation time (min)	B: Photocatalyst (g/L)	C: pH	D: Degradation efficiency (%)
1	60	1.5	10	90.6
2	90	1.0	7	90.9
3	90	1.0	7	90.9
4	40	1.0	7	84.8
5	60	0.5	10	77.1
6	90	1.84	7	100
7	120	0.5	4	58.6
8	120	0.5	10	84.9
9	90	1.0	2	63.6
10	90	0.16	7	70.4
11	60	0.5	4	67.6
12	141	1.0	7	94.4
13	120	1.5	4	98.2
14	90	1.0	7	90.9
15	60	1.5	4	90.7
16	90	1.0	7	90.9
17	120	1.5	10	100
18	90	1.0	12	97.1
19	90	1.0	7	90.9
20	90	1.0	7	90.9

The obtained data was in good agreement with the reduced quadratic model. The obtained model for predicting the degradation percentage in the coded form is:

1 $$Y=90.35+2.32A+10.32B+6.88C+2.27AB+2.33AC-4.24BC-2.2{B}^{2}-3.91{C}^{2}$$

The adequacy of the obtained model was investigated with analysis of variance (Table 4). The lack of fit which compares the error of the residuals with the net error was checked and its p-value was equal to 0.0001. Due to the fact that the appropriate p-value for this parameter should be less than 0.05, the value obtained from the current working model is within the acceptable range. Also, the regression coefficient of the model showed that 94.44% of the MB degradation variable can be obtained by the model and its magnitude is sufficient to prove the adequacy and importance of the model.

**Table 4:** ANOVA analysis.

Variable	Sum of squares	df	F-value	P-value
Model	2684.43	9	23.37	< 0.0001
A: Irradiation time	73.79	1	4.93	0.0443
B: Photocatalyst concentration	1454.94	1	97.20	< 0.0001
C: pH	646.31	1	43.18	< 0.0001
AB	41.36	1	2.76	0.1173
AC	43.29	1	2.89	0.1099
BC	144.08	1	9.63	0.0089
*B*^2^	74.76	1	4.99	0.0481
*C*^2^	228.68	1	15.28	0.0023
Lack of linear fit	149.68	5		
		R^2^ = 0.9444		

According to the results obtained in the designed model, there are two binary interactions. Regarding the binary interaction of time and photocatalyst concentration, the degradation rate increases with two factors, simultaneously. By increasing the photocatalyst concentration, the surface area and number of active surface sites were increased. And also, with more time for the degradation of pollutants, the degradation rate increases. In a similar way, in the studies of Pinheiro et al., the interaction effect of time and amount of CeO_2_/g-C_3_N_4_/Ag catalyst has been investigated simultaneously. According to their results, with the increase of photocatalyst concentration and time simultaneously, the degradation rate has been increased^18^.

**Figure 7:**
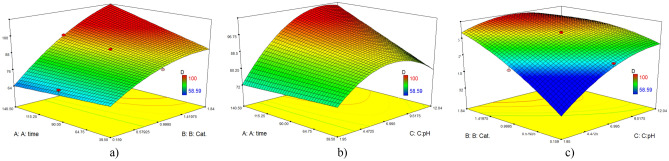
3D RSM (**a**) effect of photocatalyst concentration vs. time, (**b**) effect of pH vs. time (**c**) effect of pH vs. photocatalyst concentration.

In order to investigate the interaction effect of pH and time on the MB degradation, these two factors were investigated (Fig. 7). Assuming that other conditions are constant, with the increase in pH, the amount of pollutant adsorbed on the active surface of the photocatalyst also increases and the degradation rate increases, but there is no significant difference in the degradation rate with the change of time. In other words, pH has been a more effective factor than time in our work. Photocatalytic removal of pollutants includes two main processes: adsorption and photocatalytic oxidation. The mechanism of photocatalytic oxidation may be described by a sequence of primary reaction steps: excitation, recombination, charge trapping, generation of oxidizing species, and organic decomposition. Adsorption includes the adsorption of reactants and water. In an aqueous medium, the surface of ZIF-11/g-C_3_N_4_ 0.3 is easily hydroxylated. Cations can bond to the oxygen atoms of water molecules that are adsorbed on the surface of the photocatalyst and decompose into OH groups. In other words, pH plays the main role in the adsorption of the reactant on the surface of the photocatalyst^29^.

The effect of pH on adsorption can be explained based on the point of zero charge (pzc), where pH_pzc_ = 6.75 was obtained for ZIF-11/g-C_3_N_4_0.3. At lower pH than pH_pzc_, the photocatalyst surface has a positive charge, and at higher pH, the photocatalyst surface has a negative charge. In the case of the binary interaction of pH and photocatalyst concentration, as can be seen in the diagram, these two factors have a significant direct effect, in such a way that with the increase of each of these factors, the degradation rate increases, and with the increase of both simultaneously, higher rate of destruction can be achieved and vice versa. The reason for the higher performance of the photocatalyst in alkaline pH is the electrostatic attraction between the surface of the negatively charged photocatalyst and the cationic MB dye. Also, the increase in the concentration of OH^−^ is a factor in the increase of OH· radicals, as a result, destruction is better and the amount of pollutant is placed on the surface of the catalyst. On the other hand, with the increase in the concentration of the photocatalyst, the surface area increases and the number of active sites is more. In the studies of Fathi et al., the results obtained in the binary interaction of pH and concentration of photocatalyst (MgO/g-C_3_N_4_/zeolite) have witnessed an increase in the degradation rate by increasing both at the same time^19^.

**Figure 8:**
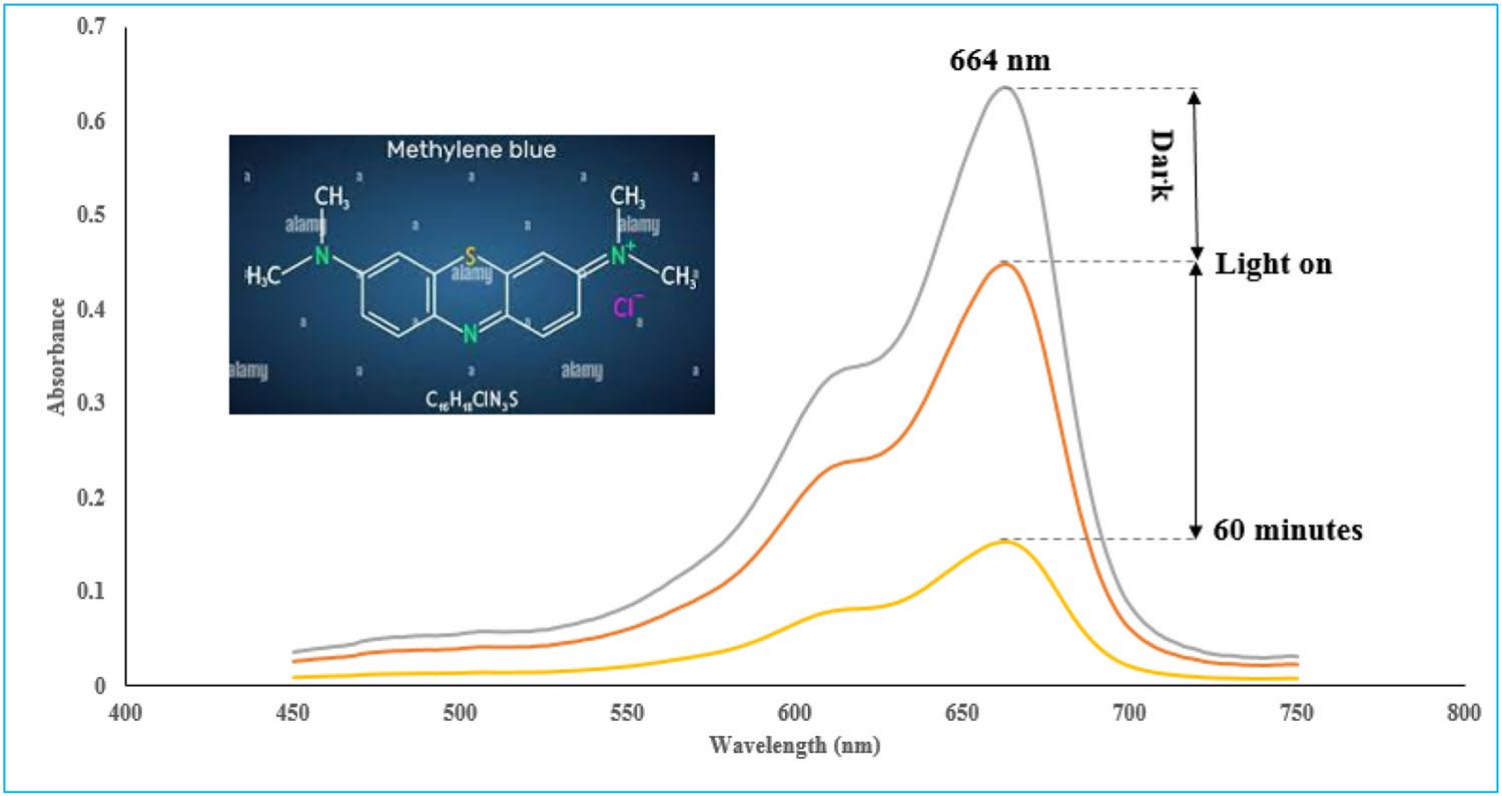
UV–Vis absorption spectra of MB using ZIF-11/g-C_3_N_4_ 0.3 in optimum operating condition.

The optimization of the operating parameters was defined by choosing the minimum irradiation time, the minimum amount of photocatalyst and the maximum achievable degradation efficiency. The conditions are presented in Table 5. UV–Vis absorption spectra of MB using ZIF-11/g-C_3_N_4_0.3 in optimum operating condition was shown in Fig. 8.

**Table 5:** Optimum operating conditions for the photocatalytic degradation of MB (10 ppm) by ZIF-11/g-C_3_N_4_0.3 under visible light.

Variable	The optimal value
A: Irradiation time (min)	60
B: Photocatalyst concentration (g/L)	0.5
C: pH	10
Degradation efficiency at 60 min (%)	77.1

In the following, the kinetics of the degradation reaction and the stability of the photocatalyst (ZIF-11/g-C_3_N_4_0.3), as well as the mechanism of charge transfer in the synthetic photocatalyst have been investigated in optimized condition (according to Table 5).

**Figure 9:**
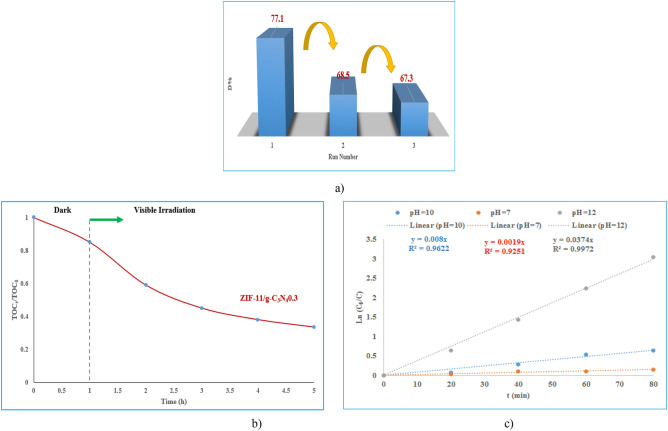
(**a**) Evaluation of reusability of ZIF-11/g-C_3_N_4_0.3 photocatalyst in MB degradation, (**b**) variation of TOC during photocatalytic degradation of MB, (**c**) Ln (C_0_/C) vs. irradiation time at different pH (MB: 10 ppm, Photocatalyst (ZIF-11/g-C_3_N_4_0.3): 0.1 g, time: 80 min, visible light: 120 W).

The stability of ZIF-11/g-C_3_N_4_0.3 photocatalyst was evaluated by repeating MB degradation experiments under visible light irradiation under the same conditions in three consecutive cycles. As shown in Fig. 9a, the photocatalytic degradation efficiency is 77.1, 68.5 and 67.3%, respectively. Photocatalytic degradation efficiency has decreased slightly after 3 cycles, which shows the stability of ZIF-11/g-C_3_N_4_0.3 sample. According to Fig. 9b, ZIF-11/g-C_3_N_4_ 0.3 was shown the highest mineralization capability, by which 67.5% of TOC was mineralized after 5 h irradiation, indicating that most of MB as well as the organic intermediates were mineralized and decomposed.

The effect of pH value on the reaction rate was investigated in three experiments by choosing pH = 7, 10, 12 and the results are shown in Fig. 9c. The kinetics of the reactions follow the pseudo-first-order model, and their rate constants are determined 0.0019, 0.008 and 0.0374 min^−1^, respectively. The results indicate that the rate of the degradation reaction is higher at alkaline pH values. As mentioned above, with the increase in pH, the concentration of hydroxide anions gradually increases, the conditions for the production of hydroxyl oxidizing radical are provided and it causes more destruction of pollutant^19,29^. As a result, it has caused an increase in the rate of degradation.

**Figure 10:**
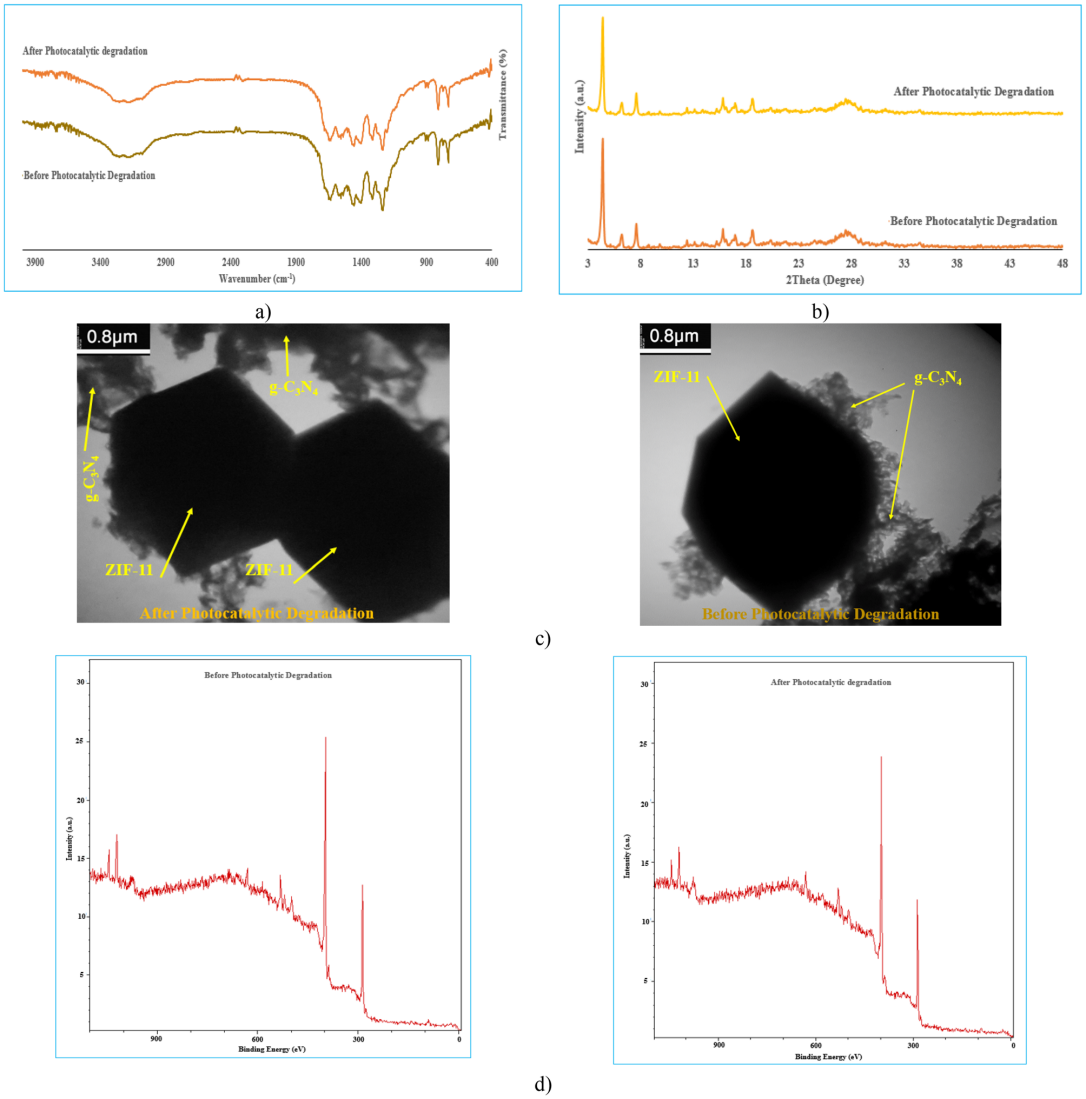
(**a**) FT-IR spectrum, (**b**) XRD pattern and (**c**) TEM images in different magnifications, (**d**) XPS survey spectra of ZIF-11/g-C_3_N_4_0.3 composite before and after MB photocatalytic degradation.

Also, in the case of the FTIR spectrum, XRD pattern, XPS spectra and TEM images obtained after the photocatalysis of MB (Fig. 10), there is no change of the positions of bands and morphologies of composite. By XPS analysis, no obvious change regarding ionic state of Zn, C and N, and chemical composition on the photocatalyst surface was detected. These results confirmed the chemical stability of the composite under photocatalytic process.

Light-induced active species play an important role in the photocatalytic process for the rate of degradation, including the hole (h^+^), superoxide anion radical (·O_2_^−^), and hydroxyl radical (·OH). Scavengers were used for three main reasons: (1) postponing the electron/hole recombination formed on the surface of the photocatalyst, (2) trapping the electron/hole or radical and increasing the performance efficiency by removing the interference effects of active compounds in the environment and increasing the role of the effective agent in pollutant decomposition and (3) playing a direct role in facilitating the breaking of the color agent bonds in the dye molecule^30–32^.

**Figure 11:**
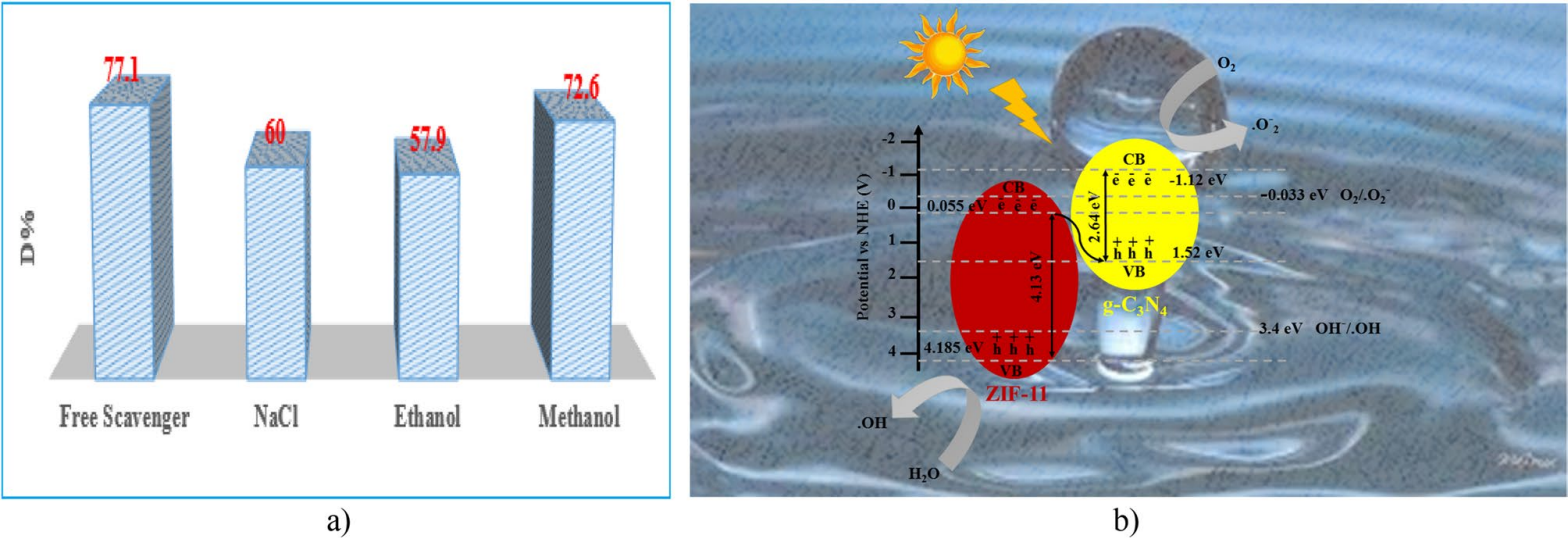
(**a**) Photocatalytic degradation performance of ZIF-11/ g-C_3_N_4_0.3 by different scavengers, (**b**) the proposed mechanism of photocatalytic process by ZIF-11/g-C_3_N_4_0.3

In this regard, methanol acts as a hydroxyl radical scavenger, ethanol was investigated as an electron absorber and sodium chloride as h^+^ Scavenger (Fig. 11a). With the addition of ethanol and sodium chloride, it can be seen that greater drop in the degradation efficiency was occurred, therefore, holes and electrons are considered as “effective primary” factors in the degradation mechanism. The results of PL analysis showed that the synthetic photocatalyst (ZIF-11/g-C_3_N_4_0.3) is suitable for the separation of charge carriers. In other hand, the results of the active species trapping experiments showed that hole and electron are effective factors in the degradation mechanism of MB.

The above topics were raised in order to further investigate the destruction mechanism. The charge carriers of ZIF-11/g-C_3_N_4_0.3 will transfer according to Fig. 11b. Electrons in the excited g-C_3_N_4_ conduction band, which have a more negative potential, reduce molecular oxygen and superoxide radical were formed to achieve efficiency. The holes in the valence band of ZIF-11, which have more positive potential, cause the creation of active hydroxyl radicals. As a result, it is a photocatalyst with a Z-scheme to produce reactive species ·O_2_^−^ and ·OH for degradation of MB. The Z-scheme mechanism can summarize the charge transfer process and the ZIF-11/g-C_3_N_4_0.3 photocatalyst creates a more effective electron migration system that is directly involved in the photocatalytic degradation of organic dyes and ultimately increases the performance of the photocatalyst.

The comparison of current results with various studies was demonstrated in Table 6. It can be concluded that ZIF-11/g-C_3_N_4_0.3 which was synthesized by simple method at room temperature, has good performance in degradation of MB by using less power of irradiation and good stability under visible light irradiation.

**Table 6:** The comparison of current results with various studies on photocatalytic degradation of MB (10 ppm).

Name	Photocatalyst	Irradiation	Time (min)	Degradation (%)	References
Ce-MOF/g-C_3_N_4_	0.25 g/L	UV light	120	96.5	^17^
Cu (II) MOF/g-C_3_N_4_	0.3 g/L (+ H_2_O_2_ (50 mM))	Visible light	45	92.3	^33^
Ag_3_PO_4_/Zr-BDC/g-C_3_N_4_	0.7 g/L	85 W visible light	240	95	^34^
Dy–Ni substituted Ca_0.5_ Pb_0.5-x_Fe_12-y_O_19_	1 g/L	Sunlight	105	85.4	^35^
g-C_3_N_4_/ZnO-W/Co_0.01_	0.05 mg	Sunlight	90	90	^36^
ZIF-11/g-C_3_N_4_0.3	0.5 g/L	120 W visible light	60	77.1	This work

## Conclusion

In this research, the zeolitic-11 imidazolate framework/graphitic carbon nitride was synthesized for the first time by a simple method and its photocatalytic performance in the degradation of MB under visible light irradiation was investigated. The resulting structure leads to the formation of a Z-scheme photocatalyst, active in visible light with a lower electron/hole recombination rate than pure ZIF-11 in the degradation of MB. The higher surface area of ​​ZIF-11/g-C_3_N_4_0.3 was comparable to pure g-C_3_N_4_. Also, the higher ability of light absorption by ZIF-11/g-C_3_N_4_0.3 indicated that g-C_3_N_4_ in the structure plays a significant role in improving the photocatalytic activity. According to the results of the statistical analysis of the effect of operational parameters, two parameters of photocatalyst concentration and pH have more effective on photocatalytic degradation efficiency. The photocatalytic degradation of MB by ZIF-11/g-C_3_N_4_0.3 under optimal operating conditions follows pseudo first-order kinetic model. The photocatalytic degradation efficiency of MB under optimal operating conditions in three consecutive cycles by ZIF-11/g-C_3_N_4_0.3 did not have a significant efficiency loss, which indicates the reusability and acceptable stability of the synthetic photocatalyst.

## Data Availability

The datasets used and/or analyzed during the current study available from the corresponding author on reasonable request.

## References

[1] Khan I, Saeed Kh, Zekker I, Zhang B, Hendi AH, Ahmad A, Ahmad Sh, Zada N, Ahmad H, Ali Shah L, Shah T, Khan I (2022). Review on methylene blue: Its properties, uses toxicity and photodegradation. Water.

[2] Farhadi H, Keramati N (2023). Investigation of kinetics, isotherms, thermodynamics and photocatalytic regeneration of exfoliated graphitic carbon nitride/zeolite as dye adsorbent. Sci. Rep..

[3] Altaf Nazir M, Najam T, Zarin K, Shahzad Kh, Sufyan Javed M, Jamshaid M, Aswad Bashir M, Ahmad Shah SSh, Ur Rehman A (2021). Enhanced adsorption removal of methyl orange from water by porous bimetallic Ni/Co MOF composite: A systematic study of adsorption kinetics. Int. J. Environ. Anal. Chem..

[4] Altaf Nazir M, Najam T, Shahzad Kh, Ahmad Wattoo M, Hussain T, Khurram Tufail M, Ahmad Shah SSh, Ur Rehman A (2022). Heterointerface engineering of water stable ZIF-8@ZIF-67: Adsorption of rhodamine B from water. Surf. Interfaces.

[5] Jamshaid M, Altaf Nazir M, Najam T, Ahmad Shah SSh, Khan HM, Rehman AU (2022). Facile synthesis of Yb^3^^+^–Zn^2^^+^ substituted M type hexaferrites: Structural, electric and photocatalytic properties under visible light for methylene blue removal. Chem. Phys. Lett..

[6] Pawan T, Rana S (2023). Structural, optical, electrical, and photocatalytic application of NiFe_2_O_4_@NiO nanocomposites for methylene blue dye. Ceram. Int..

[7] Shirzad Taghanaki N, Keramati N, Mehdipour Ghazi M (2021). Photocatalytic degradation of ethylbenzene by nano photocatalyst in aerogel form based on titania. Iran. J. Chem. Chem. Eng..

[8] Asgharian M, Mehdipour Ghazi M, Khoshandam B, Keramati N (2019). Photocatalytic degradation of Methylene Blue with synthesized rGO/ZnO/Cu. Chem. Phys. Lett..

[9] Asgharian M, Khoshandam B, Mehdipour Ghazi M, Keramati N (2021). Photocatalytic degradation of tetracycline in a stirred tank: Computational fluid dynamic modeling and data validation. React. Kinet. Mech. Catal..

[10] Hossein Zadeh M, Keramati N, Mehdipour Ghazi M (2019). Ultrasonic-assisted synthesis of new photocatalyst based on Fe-benzentricarboxylic (Fe-BTC) metal organic framework: Characterization and photocatalytic properties. J. Iran. Chem. Soc..

[11] Hossein Zadeh M, Keramati N, Mehdipour Ghazi M (2019). The effect of solvents on photocatalytic activity of Fe-BTC metal organic framework obtained via sonochemical method. Inorg. Nano-Met. Chem..

[12] Saadati F, Keramati N, Mehdipour Ghazi M (2020). Influence of parameters on the photocatalytic degradation of tetracycline in wastewater: Review. J. Water Chem. Technol..

[13] Asgharian M, Mehdipour Ghazi M, Khoshandam B, Keramati N (2020). Experimental design and RSM modeling of tetracycline photocatalytic degradation using rGO/ZnO/Cu. Desalin. Water Treat..

[14] Hong Y, Wang B, Hu Sh, Lu Sh, Wu Q, Fu M, Gu Ch, Wang Y (2023). Preparation and photocatalytic performance of Zn_2_SnO_4_/ZIF-8 nanocomposite. Ceram. Int..

[15] Roudbari R, Keramati N, Ghorbani M (2021). Porous nanocomposite based on metal-organic framework: Antibacterial activity and efficient removal of Ni(II) heavy metal ion. J. Mol. Liq..

[16] Houshyar, F. Mehdipour Ghazi, M. & Keramati, N. Optimization of operational parameters in photocatalytic degradation of methylene blue using zeolitic imidazolate framework-11 nanostructure. *Desalin. Water Treat*. (2023) **(in press)**.

[17] Durmus Z, Köferstein R, Lindenberg T, Lehmann F, Hinderberger D, Wouter Maijenburg A (2023). Preparation and characterization of Ce-MOF/g-C_3_N_4_ composites and evaluation of their photocatalytic performance. Ceram. Int..

[18] Madima N, Kefeni KK, Mishra ShB, Mishra AK (2022). TiO_2_-modified g-C_3_N_4_ nanocomposite for photocatalytic degradation of organic dyes in aqueous solution. Heliyon.

[19] Devarayapalli KC, Prabhakar Vattikuti SV, Sreekanth TVM, Soo Yoo K, Nagajyothi PC, Shim J (2020). Hydrogen production and photocatalytic activity of g-C_3_ N_4_/Co-MOF (ZIF-67) nanocomposite under visible light irradiation. Appl. Organomet. Chem..

[20] Pinheiro D, Sunaja KR, Jose A, Sankaran S, Krishna M (2021). Box–Behnken design and experimental study of ciprofloxacin degradation over Ag_2_O/CeO_2_/g-C_3_N_4_ nanocomposites. Int. J. Environ. Sci. Technol..

[21] Fathi E, Gharbani P (2021). Modeling and optimization removal of reactive Orange16 dye using MgO/g-C_3_N_4_/zeolite nanocomposite in coupling with LED and ultrasound by response surface methodology. Diam. Relat. Mater..

[22] Guo H, Gao Sh, Chai X, Shi Y, Gao J (2023). Preparation of Z-type heterojunction SiCf/g-C_3_ N_4_ composites with enhanced photocatalytic degradation of tetracycline under visible light. Appl. Organomet. Chem..

[23] Qiu J (2018). Modified metal-organic frameworks as photocatalysts. Appl. Catal. B Environ..

[24] Yuan D, Jing D, Jie Zh, Lei W, Hui W, Wei-Lin D, Guofeng G (2018). Graphite carbon nitride nanosheets decorated with ZIF-8 nanoparticles: Effects of the preparation method and their special hybrid structures on the photocatalytic performance. J. Alloys Compd..

[25] Wang Z, Zhiliang J, Guorong W, Bingzhen M (2018). Efficient hydrogen production over MOFs (ZIF-67) and g-C_3_N_4_ boosted with MoS_2_ nanoparticles. Int. J. Hydrog. Energy.

[26] Ba G, Huo T, Deng Q, Li H, Huo W (2020). Mechanochemical synthesis of nitrogen-deficient mesopore-rich polymeric carbon nitride with highly enhanced photocatalytic performance. ACS Sustain. Chem. Eng..

[27] Cheng J, Ma D, Li Sh, Qu W, Dong W (2020). Preparation of zeolitic imidazolate frameworks and their application as flame retardant and smoke suppression agent for rigid polyurethane foams. Polymers (Basel).

[28] Zhou J, Wei L, Weiquan C (2019). The synergistic effect of Ag/AgCl@ZIF-8 modified g-C_3_N_4_ composite and peroxymonosulfate for the enhanced visible-light photocatalytic degradation of levofloxacin. Sci. Total Environ..

[29] Wang X, Zhang W, Wei C, Li R, Guo J, Liu B (2020). Titanium incorporated and g-C_3_N_4_-coated NH_2_-UiO-66 for enhanced photocatalytic hydrogen evolution. Appl. Phys. A.

[30] Wenyuan H, Ning L, Xiaodong Zh, Minghong W, Liang T (2017). Metal organic framework g-C_3_N_4_/MIL-53(Fe) heterojunctions with enhanced photocatalytic activity for Cr(VI) reduction under visible light. Appl. Surf. Sci..

[31] Aeindartehran L, Siamak S, Talesh A (2021). Enhanced photocatalytic degradation of Acid Blue 1 using Ni-decorated ZnO NPs synthesized by sol-gel method: RSM optimization approach. Ceram. Int..

[32] Saadati F, Keramati N, Mehdipour Ghazi M (2016). Synthesis of nanocomposite based on Semnan natural zeolite for photocatalytic degradation of tetracycline under visible light. Adv. Environ. Technol..

[33] Zhou WJ, Ma LX, Li LY, Zha M, Li BL, Wu B, Hu ChJ (2022). Synthesis of a 3D Cu(II) MOF and its heterostructual g-C_3_N_4_ composite showing improved visible-light-driven photodegradation of organic dyes. J. Solid State Chem..

[34] Yassin JM, Taddesse AB, Sánchez-Sánchez M (2022). Sustainable synthesis of semicrystalline Zr-BDC MOF and heterostructural Ag_3_PO_4_/Zr-BDC/g-C_3_N_4_ composite for photocatalytic dye degradation. Catal. Today..

[35] Jamshaid M, UrRehman A, ParkashKuma O, Iqbal Sh, AltafNazir M, Anum M, Khan HM (2021). Design of dielectric and photocatalytic properties of Dy–Ni substituted Ca_0.5_ Pb_0.5-x_Fe_12-y_O_19_ M-type hexaferrites. J. Mater. Sci. Mater. Electron..

[36] Malik M, Ibrahim SM, Nazir MA, Tahir AA, Tufail MK, Shah SSA, Anum A, Wattoo MA, Rehman AU (2023). Engineering of a hybrid g-C_3_N_4_/ZnO-W/Co_x_ heterojunction photocatalyst for the removal of methylene blue dye. Catalysts.

